# Editorial: Chemistry, toxicity, synthesis, biological and pharmacological activities of coumarins and their derivatives: recent advances and future perspectives

**DOI:** 10.3389/fphar.2024.1430985

**Published:** 2024-05-31

**Authors:** Ahmed Olatunde, Habibu Tijjani, Adeyemi Oladapo Aremu, Abdur Rauf, Hafiz A. R. Suleria, Mohammad S. Mubarak

**Affiliations:** ^1^ Department of Medical Biochemistry, Abubakar Tafawa Balewa University, Bauchi, Nigeria; ^2^ Department of Environmental Health Science, National Open University of Nigeria, Abuja, Nigeria; ^3^ Indigenous Knowledge Systems Centre, Faculty of Natural and Agricultural Sciences, North-West University, Mmabatho, South Africa; ^4^ School of Life Sciences, College of Agriculture, Engineering and Science, University of KwaZulu-Natal, Durban, South Africa; ^5^ Department of Chemistry, University of Swabi, Swabi, Pakistan; ^6^ The University of Melbourne, Parkville, VIC, Australia; ^7^ Department of Chemistry, The University of Jordan, Amman, Jordan

**Keywords:** phytochemicals, structure-activity relationship, biological effects, toxicity profile, drug candidates

**FIGURE 1:**
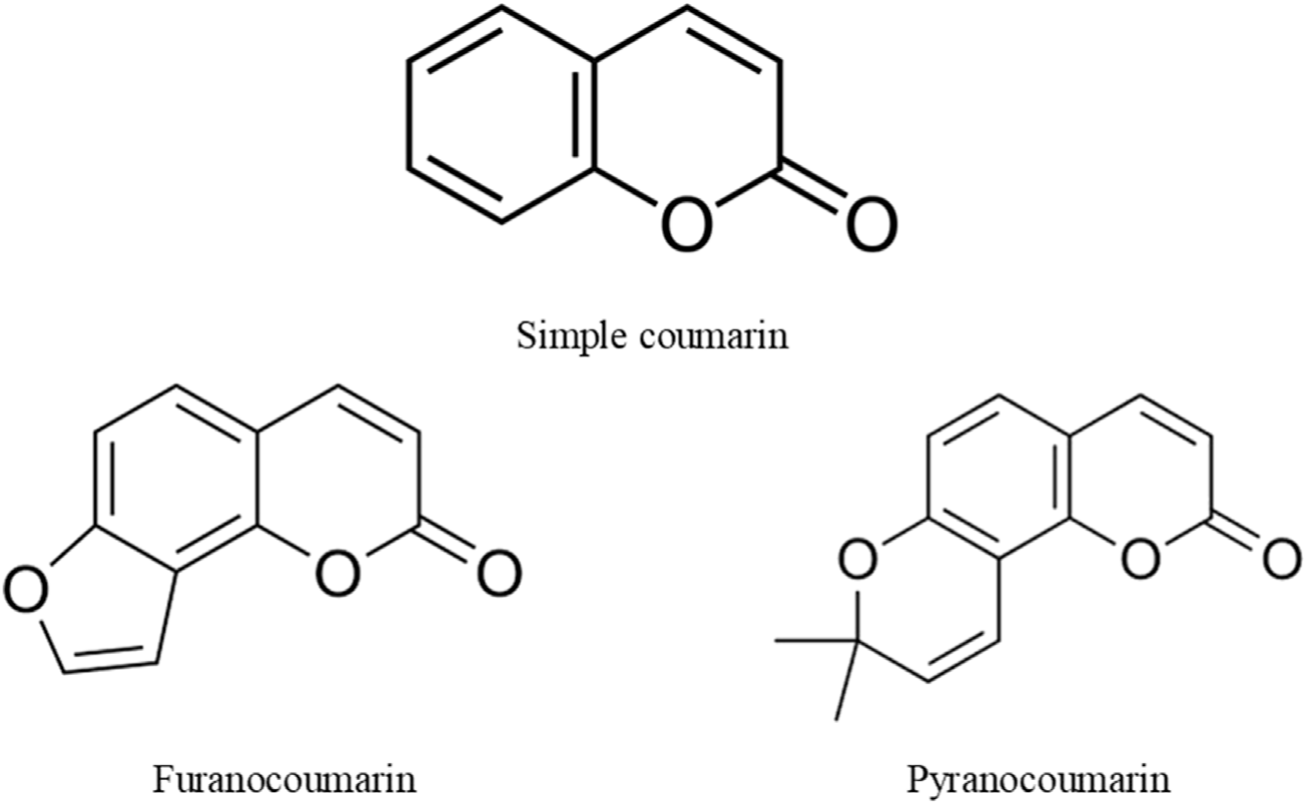
Structures of coumarin, furanocoumarin, and pyranocoumarin.

Coumarins are plant-derived compounds existing in bond form as glycosides or esters and in free form. They are found in fungi and plant parts such as roots, seeds, flowers, and fruits (Heghes et al.). The name “coumarin” was derived from *Dipteryx odorata* (Aubl.) Forsyth f. (Synonym: *Coumarouna odorata* Aubl) of the Fabaceae, a plant from which it was first isolated. Coumarins are use in foods, cosmetics and pharmaceuticals due to their pleasant smell. They are lactones of 2-coumaric acid and consist of a benzene ring fused to an *α*-pyrone ring. Based on their structural complexity, and diversity, natural coumarins are classified into various groups, including simple coumarins, furanocoumarins, and pyranocoumarin (Figure 1). However, laboratory procedures are available for synthesizing the non-natural forms of these compounds. This gives rise to many analogues of coumarins with synthetic origin. In addition, these compounds and their analogues have shown strong effectiveness in managing various diseases affecting humans (Adetunji et al., 2024; Anywar and Muhumuza). The potent health-promoting property of coumarins was linked to the versatility of their structures.

In this Research Topic, we focused on different coumarins and their analogues in terms of their pharmacological and biological activities, toxicity, sources, and classification as well as data on their synthetic forms. The Research Topic presents seven peer-reviewed articles consisting of three research papers and four reviews. These submissions entails the current research progress made on coumarins and their derivatives.

In the Research Topic, Devi et al. evaluated the acute and sub-acute toxicities of dihydro-*p*-coumaric acid obtained from the leaves of *Tithonia diversifolia* (Hemsl.), a flowering plant in the Asteraceae. The isolated dihydro-*p*-coumaric acid had no detrimental effects at all the applied doses in the acute and sub-acute toxicity tests. However, mild hepatotoxicity was recorded in alanine transaminase and aspartate transaminase, with no corresponding alteration in the histopathological examinations (Devi et al.). Furthermore, the researchers indicated that dihydro-*p*-coumaric acid is safe and can be used as a potential alternative to artificial pesticides regardless of the mild alteration that was recorded on the liver biomarker enzymes. Hence, the authors recommended the potential application of coumaric compounds as natural pesticides and as alternatives to their synthetic counterparts, due to the safety, efficacy, availability, and cost. In another study (Heghes et al.), the safety profile of natural coumarins found in nutraceuticals was explored. Despite the safety profile of these coumarins in nutraceutical products, these compounds could have some detrimental effects, especially after exposure at high concentrations. For instance, osthole (7-methoxy-8-(3-methyl-2-butenyl)-2*H*-1- benzopyran-2-one) was one of the coumaric compounds tested for acute or sub-chronic toxicity. The LD_50_ of 710 mg/kg body weight in mice was reported for the acute toxicity studies and the clinical toxicity manifestations were tremors, hyperventilation, and photophobia (Heghes et al.). Similarly, at doses of 5–50 mg/kg body weight/45 days, osthole induced a mild inflammatory process in hepatic and renal tissues, and pulmonary hemorrhage was observed in a sub-chronic toxicity study (Shokoohinia et al., 2017). Despite these mild harmful effects, the authors concluded that coumarins are still important natural compounds found in several herbs and foods with a wide range of vital biological properties, and are important for promoting health and alleviating different diseases. Moreover, the most vital detrimental effect shown by these coumarins was their mild hepatotoxicity. However, they do not promote CYP2A polymorphisms; they can be more toxic for groups presenting certain CYP2A polymorphisms (Heghes et al.).

The bioactivity and toxicity of coumarins was reviewed by Anywar and Muhumuza. The authors critically assessed the 22 coumarins existing in 15 African medicinal plants. Most of these coumarins were predominantly (19.1%) found in the leaves and frequently (46.7%) screened for anti-microbial properties. Interestingly, five (calanolide, osthole, esculetin, pseudocordatolide C, and chartreusin) of the 22 compounds exerted potent anticancer and antitumor actions. Other African medicinal plants contain coumarins that exhibited several ameliorative actions against tremor, hepato-, neuro-, and photo-toxicity, pulmonary hemorrhage, photophobia, and hyperventilation. The authors indicated that coumarins exhibit diverse biological properties and some toxic effects. In this regard, it was recommended that more toxicity studies are required to ascertain the safety of these compounds in addition to their biological efficacies (Anywar and Muhumuza).

To further highlight the wide range of biological activities of these compounds, a prominent coumarin called daphnetin, was reviewed. Daphnetin is naturally synthesized from a shikimic acid cascade, involving L-phenylalanine and L-tyrosine as the precursors for its biosynthesis (Santos-Sánchez et al., 2019). Synthetically, it can be produced from malate and pyrogallol in the presence of heat, sulphuric acid, and nitrogen. Also, the hydroxylation of umbelliferone can result in the formation of daphnetin (Wang et al., 2020). Daphnetin is isolated from *Daphne* species in the Thymelaeaceae and documented to possess several biological activities including analgesic, anti-arthritic, antipyretic, antimalarial, antioxidant, antibacterial, nephroprotective, hepatoprotective, neuroprotective, anticancer, and anti-inflammatory. In addition to these pharmacological properties, daphnetin has a promising safety profile (Javed et al.). In one of the acute toxicity studies using the bacterial reverse mutation assay, daphnetin had no genetic toxic effects. Furthermore, daphnetin was considered safe with little or no harmful effect *in vitro* and *in vivo* studies (Yu et al., 2014). Due to their broad range of biological effects, and safety profile, coumaric compounds should be utilised as a pharmaceutical scaffold or supplements in drug development. From a structure-activity relationship perspective, several derivatives of daphnetin were documented and linked to their diverse biological activities. For instance, the substitutions on C4 of daphnetin formed several derivatives, including 4-carboxymethyl daphnetin, with potent antioxidant activity. The most potent antioxidant activity of these substituted coumaric compounds was displayed by 4-carboxymethyl daphnetin (Dar et al., 2015; Xia et al., 2018).

Another interesting coumaric compound that was systematically reviewed by Gao et al. is scopoletin, where its toxicity, pharmacokinetic, and pharmacological features were highlighted (Gao et al.). This compound exerts anti-angiogenetic, antimicrobial, anti-hypertensive, anti-inflammatory, anticancer, neuroprotective, immune-modulatory, antidiabetic, and hepatoprotective effects *in vitro* and *in vivo* models. Scopoletin possesses fast absorption, extensive metabolism, and low bioavailability as demonstrated in the pharmacokinetic studies. These researchers related these pharmacokinetic features to the poor solubility of the compound in aqueous solution. In the aspect of toxicity, findings indicated that scopoletin is non-toxic to the majority of the cell types tested, suggesting that the compound will neither cause impaired performance nor treatment-related mortality at the experimental dose (Gao et al.). Although the compound exhibited a broad spectrum of biological activities, fast absorption, extensive metabolic profile, and minimal or low toxic effect, the researchers recommended more studies focusing on its cellular targets and mechanisms as well as increasing its oral bioavailability through enhancing the compound’s solubility in aqueous media.

In conclusion, this Research Topic highlights the cellular, molecular, and biochemical roles of coumarins and their derivatives against diverse diseases, both metabolic and non-metabolic, due to their vast and diverse biological properties. Additionally, the sources, synthesis, and toxicity profiles, along with the pharmacokinetics and bioavailability, including the case of scopoletin, were highlighted. However, reports from clinical trials are lacking in the Research Topic, therefore, more work is encouraged and recommended to support and confirm the documented biological activities, safety, and bioavailability of these compounds from *in vitro* and *in vivo* studies.
